# The Effect of Three Desensitizing Toothpastes on Dentinal Tubules Occlusion and on Dentin Hardness

**DOI:** 10.3390/biomedicines11092464

**Published:** 2023-09-05

**Authors:** Emilia Bologa, Simona Stoleriu, Irina Nica, Ionuț Tărăboanță, Andrei Georgescu, Ruxandra Ilinca Matei, Sorin Andrian

**Affiliations:** 1 Faculty of Dental Medicine, “Grigore T. Popa” University of Medicine and Pharmacy, 16 Universitatii Str., 700115 Iași, Romania; bologa.emilia@umfiasi.ro (E.B.); nica.irina@umfiasi.ro (I.N.); ionut-taraboanta@umfiasi.ro (I.T.); andrei.georgescu@umfiasi.ro (A.G.); sorin.andrian@umfiasi.ro (S.A.); 2 Faculty of Medicine and Pharmacy, University of Oradea, 1st December Sq., 410068 Oradea, Romania

**Keywords:** dentin hypersensitivity, dentin hardness, desensitizing agent, EDX, nanohydroxyapatite, SEM, zinc-nanohydroxyapatite

## Abstract

There are two main methods used for dentin hypersensitivity (DH) treatment: dentinal tubule occlusion and blockage of nerve activity. Dentifrices are the most common vehicles for active ingredients used for DH treatment. The aim of this study was to evaluate the efficacy of three toothpastes on dentinal tubule occlusion, mineral acquisition, and dentin hardness. Forty human dentin disks were submerged in 40% citric acid for 30 s and then exposed to tooth brushing for 2 min twice a day for 14 days using three toothpastes: Dontodent Sensitive (group 1), Dr. Wolff’s Biorepair (group 2), and Sensodyne Repair and Protect (group 3). In the control group (group 4), the samples were brushed with water. All of the samples were evaluated using scanning electron microscopy (SEM), energy-dispersive X-ray (EDX), and Vickers dentin hardness determination. On SEM images, the degree of dentinal tubule occlusion was assessed using a five-grade scale. The mean score values in groups 1–4 were 3.60 ± 0.69, 2.20 ± 0.91, 2.30 ± 1.16, and 5.00 ± 0.00, significantly higher in study groups when compared to the control group (Kruskal Wallis test *p* < 0.05). EDX evaluation showed significantly higher calcium and phosphorus concentrations in groups 1 and 3 when compared to control group d. The mean values of Vickers dentin hardness numbers in groups 1–4 were 243.03 ± 10.014, 327.38 ± 56.65, 260.29 ± 37.69, and 225.83 ± 29.93, respectively. No statistically significant results were obtained when comparing the hardness mean values in groups (Kruskal-Wallis statistical test, *p* = 0.372 > 0.05). All three toothpastes tested demonstrated significant occlusion of dentinal tubules. Dontodent Sensitive and Sensodyne Repair and Protect toothpastes enhanced the calcium and phosphorus content of the dentin surface. None of the toothpastes increased dentin hardness as a result of mineral acquisition.

## 1. Introduction

Two conditions are mandatory for dentin hypersensitivity (DH) to occur: the dentinal tubules should be exposed into the oral cavity, and the dentinal tubules should be opened toward the pulp and toward the oral cavity [1]. Many factors are involved in DH etiology. Cervical or root dentin exposure can be a result of hard or soft tissue loss (enamel wear, gingival recession, and cementum loss) [2,3]. Dentin exposure as a result of enamel wear can frequently be determined by the association of erosive wear with abrasion, attrition, and abfraction [1,4,5]. The natural smear layer that results after dentin exposure in the absence of any acidic aggression is very stable in oral conditions and closes the dentinal tubules, preventing the occurrence of DH [6]. The exposed dentin is sensitive only if the smear layer is removed and the dentinal tubules are open [5,7]. Today, it is considered that erosion initiates the process of dentinal tubules opening, which is then amplified by abrasion, attrition, or abfraction [1,8,9]. Most frequently, root dentin exposure is caused by gingival recession and is associated with DH [10]. In gingival recession, the retraction of the gingival margin apically at the enamel-cementum junction determines coronal cementum exposure at the beginning and apical cementum exposure in more advanced cases [11]. Coronal cementum is a very thin layer (16–60 μm) that can easily be removed by tooth wear or periodontal treatment, exposing the dentin layer directly to the oral cavity [12]. Frequently, gingival recession is caused by toothbrushing, occlusal loading, periodontal disease, or periodontal treatment [13,14]; the most important risk factors are a thin periodontal biotype, the absence of attached gingiva, or a thin alveolar cortical shell [15].

There are two main methods used for DH treatment: dentinal tubule occlusion and blockage of nerve activity. Sealing of the dentin surface in occlusive therapy decreases the movement of fluid inside the tubules and reduces the DH [16]. Nerve desensitization is obtained by chemical agents that suppress or modify nerve polarization. The procedures used in DH treatment can be applied at home or in the office. In non-invasive at-home procedures, the patients apply the active ingredients by using toothpastes, mouthwashes, gels, or chewing gum. In-office therapy includes non-invasive procedures like gels, foams, vanishes, dentinal adhesive application, iontophoresis, microinvasive procedures like laser therapy, and restorative methods using composite resin or glass ionomer cements [14,17]. The products used at home are available for the treatment immediately, come at a small price, and can be self-administrated. In DH associated with gingival recession, non-invasive treatment is first recommended, and then, if indicated, periodontal surgical procedures are performed.

Dentifrices are the most common vehicles for active ingredients used for DH treatment. They are preferred because of their small price, ease of use, and home application. Active ingredients such as strontium chloride, potassium nitrate, dibasic sodium citrate, formaldehyde, sodium fluoride, sodium monofluorphosphate, and stannous fluoride are included in a complex composition [18,19,20,21]. The application of fluorides seems to create a barrier by precipitating calcium fluoride crystals, which are formed especially in the inlet of the dentinal tubules. The precipitate is slowly soluble in saliva, which may explain the transitory action of this barrier [22]. Abrasive components of the dentifrices like calcium phosphate, calcium carbonate, silicate, or aluminum can determine dentin tubule obliteration by precipitation or by smear layer formation during brushing [19,20]. The multitude of products for DH treatment on the market indicates that we are far from reaching the ideal product. Choosing the method and the product for DH treatment remains the doctor’s option based on their personal experience, preferences, and knowledge.

The aims of the present study were to compare the efficacy of three commercial desensitizing toothpastes on dentinal tubule occlusion by scanning electron microscopy (SEM) evaluation of dentin mineral deposition by energy-dispersive X-ray (EDX) analysis and by dentin hardness determination. The null hypothesis was that there is no difference in tubule occlusion, mineral acquisition, or dentin hardness when the selected dentifrices were used.

## 2. Materials and Methods

**Figure 1:**
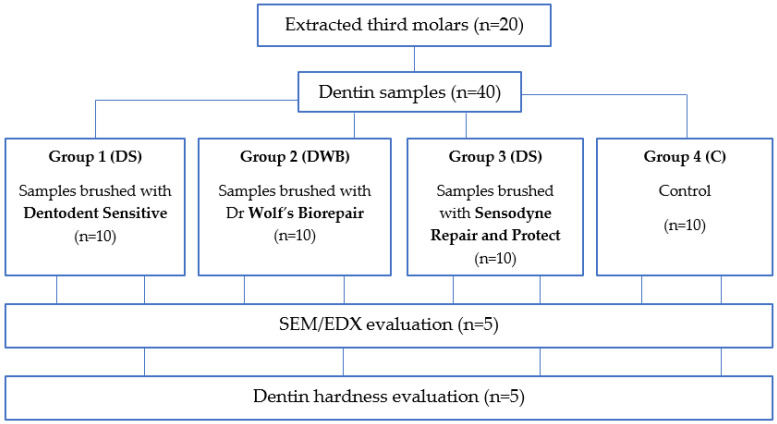
Study design.

The study design is presented in Figure 1. Details of each study step are described below.

### 2.1. Teeth Collection and Sample Preparation

The sample size was calculated using G*Power software (version 3.1.9.7., Heinrich Heine-Universität Düsseldorf, Düsseldorf, Germany). It was used with an effect size of 0.90, an alpha value of 0.05, and a power of 95%. The results estimated a total number of 40 required samples.

Twenty extracted human permanent third molars were used for this study. In order to be included in the study, the teeth should have a complete crown and present no caries, wear, cracks, or fillings. After removing soft tissues, the teeth were stored in distilled water containing 0.2% thymol until the beginning of the study [23].

For every tooth, the crown was separated from the root using a diamond disc (NTI-Kahla GmbH, Kahla, Germany) at 5000 rpm under abundant cooling water. The tooth crowns were then embedded in self-curing acrylic resin blocks (Duracryl Plus, Spofa Dental, Jičín, Czech Republic). Then, from the middle part of each crown, two dentin discs with a thickness of 1 mm were obtained by cutting the tooth perpendicular to the long axis with a diamond disc at slow speed. All the dentin sections were then polished using 600-grit silicon carbide abrasive papers for 20 s to create a uniform smear layer. To simulate the sensitive tooth model and to open the dentinal tubules, all the dentin blocks were submerged for 30 s in 40% citric acid (Cerkamed, Stalowa Wola, Poland). After that, the sections were rinsed with distilled water and introduced into an ultrasonic bath for 10 min. The resulting specimens were randomly and equally distributed into four groups. In study groups 1–3, three commercial desensitizing toothpastes were applied by brushing on dentin sample. In control group 4, the sections were brushed with water. The three toothpastes selected to be tested in the study groups were Dontodent Sensitive (DS) group 1, Dr. Wolff’s Biorepair (Dr. Kurt Wolff GmbH & Co. KG, Bielefeld, Germany) group 2 (DWB), and Sensodyne Repair, and Protect (GlaxoSmithKline, Brentford, Middlesex, UK) group 3 (SRP). The brand name, manufacturer name, and chemical composition of the tested toothpastes are presented in Table 1.

Toothpaste slurries were prepared by mixing water and toothpaste (2:1 by volume) [24]. The slurries were applied on the surface of dentin disks using a brushing machine that operates with back-and-forth movement with an amplitude of 30 mm (15 mm in each direction), a frequency of 60 cycles/minute at 1.5 Hz, and a 250 g vertical load. The application protocol was also described in a previous study [16]. The samples were brushed for 2 min twice a day for 14 days using medium-hardness bristle toothbrushes (Classic Deep Clean, Colgate-Palmolive Company, New York, NY, USA), which were changed after each brushing. After tooth brushing sessions, all the samples were rinsed under abundant deionized water and stored in artificial saliva (AFNOR NF S90–701) until the next brushing procedure. The last tooth brushing session was followed by rinsing the samples with deionized water and air drying.

### 2.2. Dentinal Tubules Occlusion by SEM Evaluation

Five samples from each group were morphologically evaluated using a scanning electron microscope (Vega Tescan LMH II, Tescan, Brno, Czech Republic), which operates at 30 kV and 15.5 WD. Ten photomicrographs of every sample were taken under 2000× magnification to evaluate the occlusion of the dentinal tubules. Two examiners, blinded to the protocol, independently evaluated the photomicrographs and assessed the dentinal tubules occlusion according to a scoring system with five grades: score 1 = occluded (100% of tubules occluded); score 2 = mostly occluded (50 to less than 100% of tubules occluded); score 3 = partially occluded (25 to less than 50% of tubules occluded); score 4 = mostly unoccluded (25 to less than 50% of tubules occluded); score 5 = unoccluded (0%, no tubule occlusion) [25]. In cases of disagreement on scoring, both examiners re-evaluated the specific image until they came to an agreement. For each sample, the final score of dentinal tubule occlusion was the average of the registered scores after ten images were evaluated.

### 2.3. Mineral Evaluation by Energy-Dispersive X-ray (EDX) Analysis

The samples evaluated using SEM were also analysed by X-ray dispersive spectroscopy. A Quantax QX2 (Bruker/Roentec, Berlin, Germany) detector was used for chemical element determination. A qualitative evaluation of the chemical elements on a selected area was performed using a P/B-ZAF database. Quantitative determination of ion concentrations (wt%) were performed in ten different areas of each dentin sample. For every sample, the ion concentrations were reported as the average value of ten determinations.

### 2.4. Dentin Hardness Evaluation

Five dentin samples from each group were submitted to surface hardness evaluation using a tribometer (CETR UMT-2, Bruker Corporation, Berlin, Germany). A Vickers-type indenter having a diamond cone with an angle of 120° and a tip with a radius of 200 μm was used for the microindentation test. The following parameters were used to obtain the indentations: a vertical force of 5 N, a speed of 0.005 mm/s, a preload time of 15 s, a charging time of 30 s, a holding time of 15 s, and a download time of 30 s. Surface hardness was automatically calculated by the software (Tribometer CETR UMT-2, Version 1.01 software, Bruker Corporation, Berlin, Germany) from the discharge slope curve and expressed in GPa.

### 2.5. Statistical Analysis

SPSS 27.0 software (SPSS Inc., Chicago, IL, USA) was used for statistical analysis. The Kolmogorov-Smirnov normality test and the non-parametric Kruskal-Wallis test were used to compare the mean scores of dentinal tubule occlusion and dentin hardness among groups. A value of 0.05 was set as a statistically significant level.

## 3. Results

### 3.1. SEM Evaluation Results

**Figure 2:**
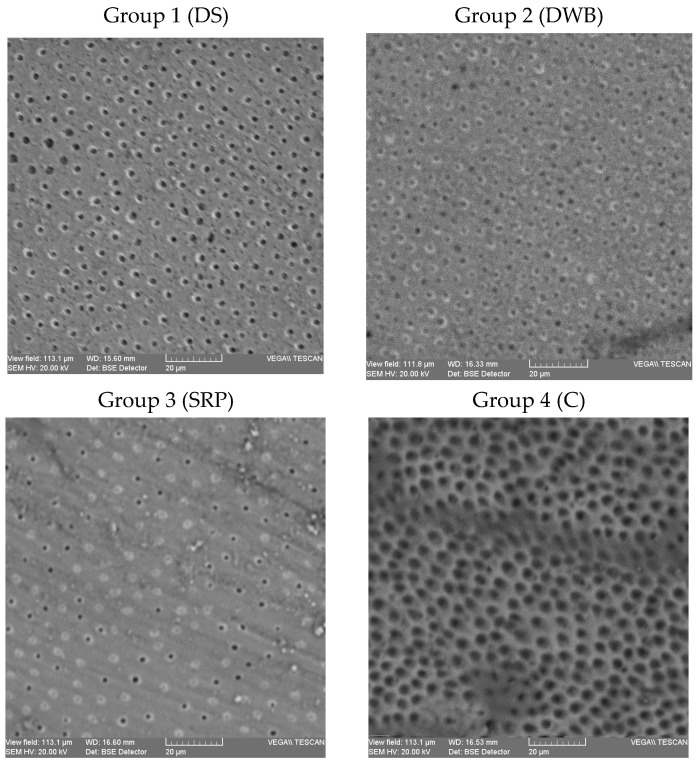
SEM micrographs of dentin samples in control and study groups. SEM micrographs of dentin samples in study groups (1–3) and control (4) at ×2000 magnification; DS—Dentodent Sensitive toothpaste; DWB—Dr. Wolff’s Biorepair toothpaste; SRP—Sensodyne Repair and Protect toothpaste; C—control, no toothpaste.

Examples of the dentin surface morphological aspects of some samples in the control and study groups are presented in Figure 2. Surface analysis of dentin samples revealed a rare area of mineral precipitation on intertubular dentin but obvious mineral deposits on tubule openings. SEM micrographs showed different degrees of dentinal tubules in groups. In group 1, the majority of the samples were evaluated with a score of 4 (70%), followed in descending order by the samples scored with 3 (20%) and the samples scored with 2 (10%). In this group, there was no sample evaluated with a score of 1 and 5. In group 2, the highest percentage of samples was scored with 2 (50%), followed by the samples scored with 1 and 3 (20%), and the samples scored with 4 (10%). No sample with a score of 5 was registered in this group. In group 3, a more homogenous distribution of scores was recorded: 20% of the samples were evaluated with scores 3 and 4, and 30% of the samples were evaluated with scores 1 and 2. There was no sample scoring 5 in this group. In group 4, all the samples were evaluated with a score of 5.

The mean values of tubule occlusion in the study and control groups are presented in Table 2. Significantly lower mean score values were registered in the study groups when compared to the control (Table 3).

### 3.2. Energy-Dispersive X-ray (EDX) Evaluation Results

EDX elemental analysis of the samples in groups showed the presence of carbon, oxygen, silicon, calcium, and phosphorus on the sample surface. In all groups, normalized weight percentages of oxygen were recorded, followed by calcium, carbon, and phosphorous (Table 4). A very low value of the ion weight percentage was registered in all study groups and in the control group. The fluorine ion was undetectable (mass concentration and normalized weight percent were lower than the values of absolute and relative error of detection). Carbon, silica, and oxygen ion concentrations were significantly lower in the control group when compared to all three study groups. Calcium and phosphorus ion concentrations were significantly higher in groups 1 and 3 when compared to the control group.

### 3.3. Dentin Hardness Test Results

The mean values of dentin surface hardness in the control and study groups are presented in Table 5. No statistically significant results were obtained when comparing the hardness values among groups (Kruskal-Wallis Chi-Square = 0.186, *p* = 0.980 > 0.05).

## 4. Discussion

In this study, we evaluated dentinal tubule occlusion by mineral deposition on the dentin surface and the changes in dentin surface hardness after using some commercial desensitizing toothpastes. All the tested toothpastes determined significant occlusion of dentinal tubules, so the null hypothesis was rejected. An efficient toothpaste used in DH treatment should present a high capacity for dentinal tubule occlusion and a good performance during intraoral acidic attacks [26]. In a systematic review of the literature, after analyzing 35 in vitro studies, Behzadi et al. concluded that desensitizing products based on bioactive glass and n-HAP are very efficient in dentin tubule occlusion [27]. Sodium calcium phosphosilicate bioactive glass (NovaMin^®^ technology) in Sensodyne Repair and Protect toothpaste serves as a reservoir for calcium and phosphate ions, resembling biological apatite [28,29]. In the toothpaste, calcium, phosphate, sodium, and silica dioxyde ions are included in an amorphous matrix [29]. When the product comes into contact with an aqueous environment (water or saliva), sodium ions will be released. That will cause the pH to increase, which in turn will favor a fast precipitation of calcium and phosphate ions in the form of a hydroxyapatite layer. Previous studies have demonstrated that this protective layer of amorphous calcium phosphate is formed one hour after contact with a simple buffer solution [30]. By scanning electron microscopy evaluation, it was concluded that bioglass determines apatite layer formation, which can occlude the dentinal tubules [31]. NovaMin^®^ technology, like pure n-Hap or Zn/n-HAP, also acts by plugging formation in dentinal tubules. The bioactive glass particles attach to the dentin-exposed collagen fibers, forming a sealant layer that continuously releases calcium and phosphate ions [32]. Other studies have mentioned that an important advantage of bioactive glasses when compared to n-Hap or Zn/n-HAP is the potential to initiate cellular migration and pulp stem cell proliferation and differentiation, which will stimulate reparative dentin formation [27]. In vitro studies have shown that NovaMin^®^ in a concentration that exceeds 3% can block at least 75% of the opened dentinal tubules after just one application and can resist frequent acidic challenges [30]. High potential for dentinal tubule occlusion of desensitizing toothpastes based on 1% n-HAP and NovaMin^®^ technology was also reported in the study of Kulal et al. (percentage of dentin tubule occlusion of 97.62% and 81.9%, respectively) [28]. Shah et al. also found an increased capacity of dentinal tubule occlusion (95.58%) when testing SHY-NM toothpaste based on NovaMin^®^ technology [33]. When compared to stanium fluoride, NovaMin^®^ products can lead to protective layer formation, despite the fact that both determine dentinal tubule occlusion [32]. Mahmoodi et al.’s transversal dentin section analysis revealed occlusive deposit formation on the dentin surface and inside the dentin tubules at different depths when stanium fluoride and NovaMin^®^ desensitizing toothpastes were used, but only the NovaMin^®^ product determined significant tubule occlusion by this protective layer formation inside the tubules.

Decreased dentin permeability was reported in the study of Wang et al. after 24 h of immersion in saliva when a product based on NovaMin^®^ technology was applied. After the acidic attack, a reduction in the deposits formed inside the dentinal tubules was observed, but the crystals were still present. After a prolonged period of bathing in artificial saliva, the dentin surface morphology changed, and a homogenous layer that completely sealed the dentinal tubules formed [26].

In our study, Dr. Wolff’s Biorepair toothpaste (active ingredient Zn/n-HAP) determined similar dentinal tubule occlusion to Sensodyne Repair and Protect toothpaste (based on NovaMin^®^ technology) and Dentodent Sensitive toothpaste (active ingredient n-HAP). In a clinical study, Gopinath et al. also demonstrated that n-HAP toothpaste had the same efficiency in DH reduction as toothpaste based on NovaMin^®^ technology [34]. The results of our study are also in agreement with the study of Poggio et al., which evaluated the capacity of desensitizing toothpastes to prevent dental erosion caused by an acidic beverage and demonstrated that Biorepair Plus-Total Protection (active ingredient Zn substituted n-HAP-MicroRepair^®^ technology) and Sensodyne Repair and Protect (NovaMin^®^ technology) presented high potential for dentinal tubule occlusion [35]. The same study demonstrated the remineralization capacity of both products, with the minerals being integrated into the collagen network after acidic attack.

In our study, all the tested toothpastes had the same efficacy on dentinal tubule occlusion. The same result was also reported by Arnold et al. after analyzing different desensitizing toothpastes [36]. On the contrary, other studies have concluded that n-HAP toothpaste (nanoXIM^®^ technology) was significantly more efficient in dental tubule occlusion when compared to Sensodyne Repair & Protect toothpaste, with 66.13% of the tubules being occluded. Excellent results after seven days of using n-HAP toothpastes were also reported in Pei et al.’s study [37]. The higher efficiency of Dr. Wolff’s Biorepair and Dontodent toothpastes in Pei’s study when compared to the results of the present study can be explained by different experimental conditions (1% citric acid application for 20 s in Pei’s study for dentin tubule occlusion). Lower concentrations and decreased time of acid action cause a reduced opening of the dentinal tubules and facilitate a more rapid and complete occlusion of the tubules when desensitizing toothpastes are applied.

Excluding calcium phosphosilicate, Sensodyne Repair and Protect toothpaste contains the active ingredient stannous fluoride. For many years, fluoride products were considered the gold standard in tooth remineralization and tooth protection against acidic attacks, but the action of fluorides in desensitizing DH is still unclear. In a review of the literature, Fiorillo L. et al. highlighted the effects of stannous fluoride compounds on dental hard tissues [38]. Previous studies demonstrated similar occlusion of dentinal tubules when using toothpastes containing 0.454% stannous fluoride and 0.76% sodium monofluorophosphate, but a greater degree of occlusion for stannous fluoride toothpaste after acidic attack [10]. Another toothpaste of the three tested products in our study, Dentodent Sensitive, has an active ingredient that is a fluoride compound (sodium fluoride). It was demonstrated that fluorides may increase the mineralization of hydroxyapatite [39] and may enhance hydroxyapatite crystal formation within the dentine tubules, which blocks dentinal fluid movement and reduces pain.

EDX analysis of the dentin surface showed increased carbon ion concentrations and decreased calcium and phosphorus ion concentrations in the control group. The dentin after desensitizing toothpaste application presented decreased carbon and increased calcium and phosphorus ion concentrations. These results confirm the hypothesis of calcium and phosphate ions being released from n-HAP and NovaMin^®^ products. Calcium phosphate layer formation as a result of 5% and 7.5% NovaMin^®^ product application has been reported in previous studies [40]. In this study, the increased calcium and phosphate ion concentrations on the dentin surface did not increase dentin hardness. Except the density of the mineral phase, there are other factors that can influence dentin hardness: the location, density, and direction of the dentinal tubules or the direction of the collagen fibers [41]. Other studies reported an increased dentin hardness when compared to demineralized and sound dentin after NovaMin^®^ product application, but the research protocol was different [40].

One limitation of this study is the evaluation of dentinal tubule occlusion only by mineral deposition on the dentin surface assessment and not by the investigation of mineral penetration in tubule depth, which can allow conclusions regarding only the short-term efficacy of the products used for DH treatment. Another limitation is the use of a minimum number of dentin samples in the groups. In our study, dentin samples were obtained from the middle part of the tooth crown. There can be differences in dentinal tubule orientation and tubule diameter in this area when compared to cervical coronal or root dentin. Samples bathed in artificial saliva between toothbrushing sessions were free of acidic challenges. Future long-term research and comparative clinical-based studies must be performed to certify the efficacy of using these products for pain relief and dentin mineralization.

## 5. Conclusions

Within the limitations of this in vitro study, the three tested toothpastes based on n-HAP, Zn/n-HAP, and NovaMin^®^ technology determined the occlusion of dentinal tubules. When used twice a day for fourteen days, Dontodent Sensitive and Sensodyne Repair and Protect toothpastes increased the calcium and phosphorus ion content of the dentin surface. None of the toothpastes increased dentin surface hardness as a result of mineral (calcium and phosphate ions) acquisition. Due to their capacity for dentinal tubule blocking, the tested products can be efficiently used in immediate DH treatment.

## Figures and Tables

**Table 1:** Brand name, manufacturer and chemical composition of the tested toothpastes.

Materials’ Brand Name	Manufacturer	Composition
Dontodent Sensitive	DM Drogeria Markt, Karlsruhe, Germany	Hydroxyapatite, Sodium Fluoride, Tetrapotassium pyrophosphate, Aqua, Sorbitol, Propylene Glycol, Glycerin, Silica, Aroma, Cellulose Gum, Sodium C14–16 Olefin Sulfonate, Sodium Cocoyl Isethionate, Sodium Saccharin, Menthol, Eucalyptol, Limonene, CI 77891
Dr. Wolff’s Biorepair	Dr. Kurt Wolff GmbH & Co. KG, Bielefeld, Germany	Zinc Hydroxyapatite, Aqua, Hydrated Silica, Glycerin, Sorbitol, Silica, Aroma, Cellulose Gum, Sodium Myristoyl Sarcosinate, Sodium Methyl Cocoyl Taurate, Tetrapotassium Pyrophosphate, Zinc Pca, Sodium Saccharin, Phenoxyethanol, Benzyl Alcohol, Propylparaben, Methylparaben, Citric Acid, Sodium Benzoate.
Sensodyne Repair and Protect	GlaxoSmithKline, Brentford, Middlesex, UK	Calcium sodium phosphosilicate, Stannous fluoride, Glycerin, PEG-8, hydrated silica, pentasodium triphosphate, sodium lauryl sulfate, flavour, titanium dioxide, polyacrylic acid, cocamidopropyl betaine, sodium saccharin

**Table 2:** The scores of dentinal tubule occlusion in control and study groups (mean value ± standard deviation).

	Group 1	Group 2	Group 3	Group 4
Mean score value ± SD	3.60 ± 0.69	2.20 ± 0.91	2.30 ± 1.16	5.00 ± 0.00

**Table 3:** Kruskal-Wallis test result of comparison of the mean values of dentinal tubule occlusion in control and study groups.

Group	Compared With	Standard Error	Significance
Group 1 (DS)	Group 2 (DWB)	5.103	0.216
	Group 3 (SRP)	5.103	0.321
	Group 4 (C)	5.103	0.048
Group 2 (DWB)	Group 3 (SRP)	5.103	1.000
	Group 4 (C)	5.103	0.000
Group 3 (SRP)	Group 4 (C)	5.103	0.000

DS—Dentodent Sensitive toothpaste; DWB—Dr. Wolff’s Biorepair toothpaste; SRP—Sensodyne Repair and Protect toothpaste; C—control, no toothpaste.

**Table 4:** Ion concentrations (wt%) in control and study groups (mean values ± standard deviation).

	Mean Value of Ion Concentration (wt%) ± Standard Deviation			
Ion	Group 1	Group 2	Group 3	Group 4
Calcium	23.657 ± 5.016 ^A^	20.352 ±7.373 ^C^	24.608 ± 3.738 ^A^	17.417 ± 2.381 ^B^
Phosphorous	13.320 ± 3.323 ^A^	11.351 ± 4.979 ^C^	13.202 ± 2.910 ^A^	8.983 ± 1.919 ^B^
Carbon	14.135 ± 6.920 ^A^	16.456 ± 9.109 ^A^	11.910 ± 7.410 ^A^	35.212 ± 3.712 ^B^
Oxygen	46.057 ±1.502 ^A^	48.137 ± 4.631 ^A^	47.581 ± 2.894 ^A^	38.089 ± 2.040 ^B^
Silicon	2.828 ± 0.838 ^A^	3.702 ± 1.945 ^A^	2.696 ± 0.980 ^A^	0.296 ± 0.200 ^B^

The same capital letter in rows represent no statistically significant differences among groups (ANOVA test, *p* > 0.05, post hoc LSD test, *p* > 0.05).

**Table 5:** Dentin hardness (GPa) in control and study groups (mean values ± standard deviation).

	Group 1	Group 2	Group 3	Group 4
Mean hardness value ± SD	243.033 ± 100.147 ^A^	327.382 ± 376.653 ^A^	260.299 ± 157.697 ^A^	225.803 ± 89.934 ^A^

The same capital letter represents no statistically significant differences among groups (Kruskal-Wallis test, *p* > 0.05).

## Data Availability

All the data presented in this study are available within the article.
